# Restored perfusion and reduced inflammation in the infarcted heart after grafting stem cells with a hyaluronan-based scaffold

**DOI:** 10.1111/jcmm.12039

**Published:** 2013-03-11

**Authors:** Claudio Muscari, Francesca Bonafè, Sofia Martin-Suarez, Simond Valgimigli, Sabrina Valente, Emanuela Fiumana, Federico Fiorelli, Giuseppe Rubini, Carlo Guarnieri, Claudio Marcello Caldarera, Ombretta Capitani, Giorgio Arpesella, Gianandrea Pasquinelli

**Affiliations:** a Department of Biochemistry, University of BolognaBologna, Italy; b Department of Cardiovascular Medicine, University of BolognaBologna, Italy; c Department of Medical and Veterinary Science, University of BolognaBologna, Italy; d Department of Radiological and Histocytopathological Sciences, Division of Pathology, University of BolognaBologna, Italy; e Ultravet DiagnosticBologna, Italy; f National Institute for Cardiovascular Research (INRC)Bologna, Italy

**Keywords:** tissue engineering, woven fabric, hyaluronan-based scaffold, mesenchymal stem cell, heart perfusion, myocardial infarction

## Abstract

The aim of this study is to investigate the blood perfusion and the inflammatory response of the myocardial infarct area after transplanting a hyaluronan-based scaffold (HYAFF®11) with bone marrow mesenchymal stem cells (MSCs). Nine-week-old female pigs were subjected to a permanent left anterior descending coronary artery ligation for 4 weeks. According to the kind of the graft, the swine subjected to myocardial infarction were divided into the HYAFF®11, MSCs, HYAFF®11/MSCs and untreated groups. The animals were killed 8 weeks after coronary ligation. Scar perfusion, evaluated by Contrast Enhanced Ultrasound echography, was doubled in the HYAFF®11/MSCs group and was comparable with the perfusion of the healthy, non-infarcted hearts. The inflammation score of the MSCs and HYAFF®11/MSCs groups was near null, revealing the role of the grafted MSCs in attenuating the cell infiltration, but not the foreign reaction strictly localized around the fibres of the scaffold. Apart from the inflammatory response, the native tissue positively interacted with the HYAFF®11/MSCs construct modifying the extracellular matrix with a reduced presence of collagene and increased amount of proteoglycans. The border-zone cardiomyocytes also reacted favourably to the graft as a lower degree of cellular damage was found. This study demonstrates that the transplantation in the myocardial infarct area of autologous MSCs supported by a hyaluronan-based scaffold restores blood perfusion and almost completely abolishes the inflammatory process following an infarction. These beneficial effects are superior to those obtained after grafting only the scaffold or MSCs, suggesting that a synergic action was achieved using the cell-integrated polymer construct.

## Introduction

To date, the research devoted to the therapy of the myocardial infarction (MI) with mesenchymal stem/stromal cells (MSCs) had substantially failed their initial purpose 1–3. The ambitious end-point of substituting the necrotic myocardium with a healthy native tissue is far from being achieved. A more realistic and as much useful aim could be the prevention of the pathological remodelling of the heart by improving the structure of the myocardial scar 4. It is known that the fibrotic, post-necrotic, myocardium is represented by a thin, weakened region that is unable to properly withstand pressure and volume load. As a matter of fact, the intraventricular pressure progressively stretches either the scar or the neighbouring cardiac tissue, leading to the dilation of the ventricular chambers and, consequently, contractile heart failure 5. Beneficial effects can be obtained by increasing blood flow in the fibrotic region to improve the metabolism of the ischaemic area 6. Several studies suggest that MSCs can stimulate the vascularization of the infarcted myocardium 7–9. In particular, the MSC-mediated neoangiogenesis results in decreased apoptosis of hypertrophied myocytes in the peri-infarct region, long-term salvage and survival of viable myocardium, reduction in collagen deposition and sustained improvement in cardiac function 10. MSCs have been often grafted in the infarct area either alone 11, 12 or with scaffolds aimed at limiting their death and clearance from the site of transplantation 1. Various biomimetic polymers, *i.e*. those based on collagen, matrigel, fibrin, alginate or other natural fibres, have been already transplanted in models of MI trying to regenerate the infarcted heart 13. This study depicts a method used for the first time because a hyaluronan-based solid polymer was transplanted in pig hearts to address strategies of cell therapy and cardiac tissue engineering. Indeed, hyaluronate possesses many peculiar characteristics that could be useful for cardiac repairing as: (i) it adheres tenaciously to MSCs because of the specific interaction with the highly expressed receptor CD44 14, (ii) its small fragments which are produced by hyaluronidase and/or chemical agents are angiogenic 15 and (iii) its ester derivatives can be formulated as knitted scaffolds with elastic properties and resistance to stretch useful for contractile tissue repair 16, 17.

Campoccia *et al*. 18 extensively reviewed the key features of the hyaluronan-based knitted scaffold (HYAFF®11) used in this study. HYAFF®11 is derived through the total esterification of sodium hyaluronate (80–200 kD) with benzyl alcohol on the free carboxyl groups of glucuronic acid. Using an aqueous environment, *in vitro* studies demonstrated that the spontaneous hydrolysis of the ester bonds of HYAFF®11 requires about 2 months and that the progressive loss of esterification induces the polymer to become more hydrated and soluble. In contrast, the hyaluronan-ester backbone chain appears to be more stable than the ester bonds. With respect to cytotoxicity, HYAFF®11 appears to be a cytocompatible polymer, as proved not only by the conventional cytotoxicity screening procedures but also by extensive experimentation with many different cell types. HYAFF®11 does not significantly alter the *in vitro* neutrophil morphology or locomotion, or the level of macrophage metabolic activation as would be expected with a degrading material. HYAFF®11 used in implantation studies also appears to be an inert polymer, which gives minimal response in the first month following implantation. A macrophage-mediated response resulting from polymer degradation is usually observable after 3 months. However, such a response is mostly confined to the site where the material is implanted. Additional *in vivo* studies indicate that the material disappears about 4 months after implantation. No evidence of toxicity was detected during a 1-year study following implantation. For tissue engineering applications, the non-woven structured materials made of HYAFF®11 allow good cell adhesion and viability. An advantage of HYAFF®11-based scaffolds is good cell adhesiveness even in the absence of any coating or surface conditioning, a treatment that is often required by other widely used support matrices such as those made of polyglycolic and polylactic. The total benzyl esters of hyaluronic acid are sufficiently stable in aqueous solution to allow incubation with cells for over 3 weeks. Under *in vitro* cell culture conditions the material maintains its structural integrity, can be handled easily, and does not contract as some collagen-based materials do.

Thus, the main purpose of this proof-of-concept study was to investigate whether the transplantation of autologous MSCs with a hyaluronan-based knitted scaffold was able to induce neoangiogenesis and histological modifications to the infarcted myocardium and restore the blood perfusion in the fibrous scar.

## Materials and methods

### Animal preparation and general surgery

All procedures were performed in compliance with the relevant European laws and institutional guidelines and approved by the appropriate institutional committees. Forty-nine-week-old crossbreed female swine were stabulated. They weighted about 30 kg, and 8 weeks later, at the time of euthanasia, they had reached a body weight of approximately 45 kg. Before the surgical procedure, the animals were sedated with the intramuscular injection of a zolazepam/tiletamine admixture (Zoletil, Virbac). A double vascular access was then obtained *via* auricular veins through two 19G over-the-needle catheters. General anaesthesia was induced by the slow intravenous administration of propofol (Propovet, Esteve). The swine were then intubated and anaesthetized with isoflurane (Isoflo, Abbott) delivered through a circle system. Mechanical ventilation was initiated and maintained throughout the surgical operation. Analgesia was provided by a continuous intravenous infusion of fentanyl (Fentanest, Pfizer) and lidocaine (Lidocaina 2%, Fort Dodge), and neuromuscular blockade was obtained by administration of atracurium besylate (Tracrium, Glaxo Smith Kline). After surgery the animals were moved to a quiet and warm environment and left to recover. Analgesia was provided by administering buprenorphine (Temgesic, Schering-Plough) every 6 hrs. Routine antibiotic therapy was also administered. Before every contrast enhanced ultrasonographic examination, the animals were sedated as described above and a single vascular access obtained. A flow-by method was then used to administer 100% oxygen. Just after the last ultrasound examination, the animals were killed: sedation was deepened to total anaesthesia by the intravenous administration of thiopental (Pentothal Sodium, Intervet). A lethal overdose of potassium chloride was finally given by the same route.

### Isolation and primary expansion of MSCs

With the animal under general anaesthesia, 50 ml of bone marrow was aspirated from the iliac crest of each pig and then transferred into 50 ml of α-modified Eagle's medium (α-MEM) containing 20% foetal bovine serum (FBS), 0.1 μg/ml L-glutamine, 100 U/ml penicillin, 100 U/ml streptomycin and 250 μg/ml amphotericin B. Cells were filtered through a 70 μm nylon filter and plated into a 75 cm^2^ flask. They grew in complete α-MEM at 37°C and 5% CO_2_ for 3 days, then the medium was replaced and the adherent cells expanded for further 18 days, to reach passage 3. These adherence-selected cells, namely MSCs, were washed with phosphate-buffered saline (PBS) and detached by incubation with 0.25% trypsin and 0.1% EDTA for 3 min. at 37°C. At passage 3, the differential potential of MSCs was not modified, and the risk of genetic and/or epigenetic abnormalities was not present 19. The complete medium was added to inactivate trypsin activity. MSCs were centrifuged at 450 × *g* for 10 min., resuspended in a complete medium and counted in duplicate using a Burker haemocytometer 20.

### Immunofluorescence detection and flow cytometry analysis

Mesenchymal stem cells were transferred into 12-well plates, fixed with 3% paraformaldehyde in PBS at room temperature for 15 min. and rinsed twice with PBS after blocking with 4% bovine serum albumin (BSA) dissolved in 0.2% tween-20/PBS. Primary mouse monoclonal antibodies against pig cross-reacting CD90 (Thy-1), 1:100 (Pharmingen, San Diego, CA, USA), pig CD11 1:50 (Serotec, Oxford, UK), pig CD31 1:10 (Serotec) and pig mononuclear phagocyte factor 1:50 (MPF, Pharmingen) were used to characterized the pig MSC phenotype. The antibodies were diluted in a blocking buffer for 1 hr at room temperature and washed three times in PBS. The secondary Cy3-conjugated polyclonal anti-mouse antibody (Sigma-Aldrich, St. Louis, MO, USA) was diluted 1:2000 in a blocking buffer and washed three times in PBS. Nuclei were stained with DAPI diluted 1:1000 for 15 min. and washed three times in PBS. The samples were observed with the IX50-Olympus inverted microscopy (Olympus, Tokyo, Japan). Negative controls were obtained only with the Cy3-conjugated secondary antibody.

The presence of CD90, CD31 and CD11 was then investigated by flow cytometry analysis using the above-mentioned primary antibodies and the secondary Cy3-conjugated polyclonal anti-mouse antibody. An Epics Elite flow cytometer (Beckman Coulter, Fullerton, CA, USA) was used, equipped with a 15-mW argon ion laser tuned to 488 nm, and red fluorescence was collected at 605 nm.

Further antigenic and functional features of pig bone marrow MSCs are described in our previous study 21.

### Cell seeding and proliferation on the scaffold

Mesenchymal stem cells were seeded on a squared (3 cm × 3 cm × 0.5 mm) HYAFF®11 (Fidia Advanced Biopolymers, Abano Terme, Italy). Before cell seeding, HYAFF®11 was incubated for 1 day in the above-mentioned cell culture medium. The trypan blue exclusion staining method was used to quantify the adhesion of viable MSCs to the biopolymer. The number of cells that adhered to the fibres was calculated 24 hrs after cell seeding through a Bürker haemocytometry chamber, by subtracting the number of cells attached on the well bottom and those still in suspension from the total amount of seeded cells:


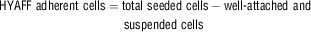


Mesenchymal stem cells were seeded at three different densities (0.2×, 0.5×, 1.0× 10^6^ cells/cm^2^), expanded for 1 week, and the viable MSCs were identified by fluorescence microscopy through the vital dye carboxyfluorescein diacetate succinimidyl ester (Vybrant CFDASE Cell Tracer Kit, Molecular Probes, Eugene, Oregon, USA).

The MSC viability within the scaffold was also quantified by the 3-(4,5-dimethylthiazol-2-yl)-2,5-diphenyltetrazolium bromide assay (MTT; Sigma-Aldrich) as previously reported, assuming the correlation between MTT concentration and living MSCs 22. Briefly, the HYAFF®11/MSCs construct was washed with PBS, transferred into a well containing 1 ml of MTT solution (1 mg/ml) and incubated for 3 hrs at 37°C. The construct was then put into an Eppendorf tube containing 1 ml of solubilization solution (0.01 M HCl in isopropanol) and vortexed for 5 min. This provoked the release of MTT whose concentration in the reduced form was representative of the amount of viable cells. The samples were centrifuged at 15,000 × *g* for 5 min. and the level of the reduced MTT in the supernatants was read at 595 nm using a multiwell spectrophotometer (Victor 2 microplate reader, Perkin Elmer, Wellesley, MA, USA). Then, the viable MSCs cultured within the biomaterial were quantified using a calibration curve relating known amounts of MSCs, grown on HYAFF®11 at different densities, with the corresponding MTT values.

To identify the engrafted MSCs in the recipient myocardium, the MSCs were labelled with 0.4% bromodeoxyuridine (BrdU; Pharmingen) 3 days before transplantation.

The MSCs adhering to the scaffold were also investigated with regard to their ability to produce VEGF compared with the MSCs grown on plastic or in suspension. The concentration of the released VEGF was measured 3 days after cell seeding using an ELISA kit (PeproTech, Rocky Hill, NJ, USA) and revealed by the Victor 2 multiwell spectrophotometer.

#### Scanning electron microscopy (SEM)

Mesenchymal stem cells cultured on HYAFF®11 were processed for SEM 7 days after cell seeding. Following fixation, the samples were dehydrated, critical point dried, mounted on aluminium stubs and then coated with a thin layer of gold using a sputtering device. The samples were observed in a Philips 505 scanning electron microscope at 10 kV 23.

#### Groups of graft transplantation

The swine were comprised into five groups:

1. Untreated infarct hearts (Ctr infarcted group; *n* = 7).
2. HYAFF®11 grafted in the infarcted hearts (HYAFF®11 group; *n* = 6).
3. MSCs grafted in the infarcted hearts (MSCs group; *n* = 6).
4. HYAFF®11 + MSCs grafted in the infarcted hearts (HYAFF®11/MSCs group; *n* = 6).
5. Untreated non-infarct hearts (Ctr non-infarcted group; *n* = 5).

#### Model of myocardial infarction

A first operation was performed through a left thoracotomy at the fourth to fifth intercostal space. The animal was placed on right lateral decubitus. Complete antisepsis was performed and a sterile surgical field was placed. A wide incision was performed and all muscle layers dissected. Once the pleural space was open, the left lung was gently excluded by compression to expose the pericardial sac. Pericardiotomy was achieved and lateral surface of the heart perfectly visualized. Then, the apex of the heart was gently exposed. The heart was progressively lifted with the interposition of wet gauzes. The permanent occlusion of the left anterior descending (LAD) coronary artery and its diagonal branch was preceded by ischaemic preconditioning. The arteries were intermittently legated with a tourniquet and alternated with progressively shorter periods of reperfusion, until their occlusion was definitive. Before the thorax closure, to reduce adhesions, we applied topically cortisone and a Covidien Vascuseal™, a synthetic and flexible sealant. After previous positioning of a drain, the thorax was closed. The animal was awakened and closely monitored for 24 hrs.

Four weeks after coronary ligation a second operation was performed to implant the graft. To avoid the adhesions provoked during the first operation, the second operation was performed with a right thoracotomy. MSCs were given by intramyocardial injections using a syringe, whereas HYAFF®11 or HYAFF®11 plus MSCs were grafted into a sub-epicardial pocket. In the groups treated with cells (MSCs and HYAFF®11/MSCs groups), we used autologous MSCs; hence, each pig received its own MSCs. For a better comparison with the effects caused by the transplantation of HYAFF®11, which covered most of the infarct region, MSCs were injected into the infarct zone rather than along the border zone.

The scaffold, with or without MSCs, was positioned inside the pocket and the myocardial flap closed and fixed above. We preferred to put the scaffold into an epicardial pocket rather than on the ventricular surface to avoid the laceration of the edge of the patch and related supported MSCs, which could result from the use of suture stitching. Moreover, by grafting the construct into a pouch it was not exposed to the atmosphere of the thoracic cavity as this could lead to dryness and early death of the cells. Just after the first and second operation, the animals were treated with amoxicillina depot (Neo Vet-Cillin LA®), BUP (buprenorfina, Temgesic®) and with amoxicillina, clavulanic acid (Synulox®) and enrofloxacine (Baytril®) for an additional 5 days. The animals were killed 8 weeks after the coronary ligation.

In the pigs subjected to myocardial infarction, the mortality was 35% and was equally distributed in each group of graft transplantation.

### Contrast-enhanced ultrasound echography (CEUS)

Contrast-enhanced ultrasound echography is a non-invasive imaging technique for quantifying tissue perfusion with microbubble contrast agents. Microbubbles of sulphur esafluoride (SonoVue, Bracco, Italy) were suspended in an isotonic solution with a viscosity similar to that of plasma 24. The average diameter of the microbubbles was 2.5 μm to allow the contrast agent to overcome the lung barrier and to dim the healthy left ventricle in a uniform manner. In humans, this analysis has proved to be effective for the study of myocardial perfusion 25. A good correlation between the blood flow measured by CEUS and a gold standard, such as microspheres, was demonstrated 26. A bolus (5 ml) of the contrast agent was injected into the femoral vein and the cardiac perfusion analysed after a few minutes. The region of interest (ROI) was drawn in the third part of the left ventricle, and included the cardiac apex. The ischaemic area was recognized as a less perfused region relative to the opposite region, which was closer to the base of the heart. The echo-contrast images of the heart were video-recorded several times for 1 min. each and then analysed by dedicated software (Qontrast 4.00; Esaote, Florence, Italy). The levels of signal intensity were measured and plotted as diagrams in 1 min. periods and used to study the rate of myocardial perfusion.

### Histochemistry and immunohistochemistry

Eight weeks after coronary ligation, the hearts were blocked in diastole with a solution of 15% KCl and rapidly excised. Cardiac cavities were rinsed with PBS to remove blood and clots. Then the hearts were fixed with 10% formalin for 24 hrs and cut into three gross pieces: apex, middle portion and base that were fixed in formalin for additional 72 hrs and then subjected to final sampling. Tissue samples were dehydrated at increasing alcohol concentrations, and embedded in paraffin. Five-μm-thick serial sections were stained with haematoxylin and eosin, Sirius Red and Alcian Blue at pH 2.5 along with periodic acid-Schiff (PAS) to assess, respectively, cardiac tissue morphology, extracellular matrix collagen and glycosaminoglycan deposition. Additional 5-μm-thick tissue sections were used for immunohistochemistry. Endogenous peroxidase activity was blocked with 3% H_2_O_2_ for 10 min. at room temperature and the antigens retrieved in a microwave for 10 min. at full power in a 10 mM citrate buffer, pH 6.0. Non-specific bindings were blocked with 10% goat serum for 45 min. Then, the slides were incubated with the primary mouse monoclonal antibody anti-BrdU (BioGenex, San Ramon, CA, USA), or anti–von Willebrand Factor (vWF, 1:200, Abcam, Cambridge, UK), or anti-CD3 (1:100; NeoMarker, Fremont, CA, USA) and identified by the NovoLinkTM Polymer Detection System procedure (Novocastra, New Castle, UK) according to the manufacturer's instructions. The circular/oval structures lined by vWF^+^ cells were identified as capillaries when their diameter was less than 20 μm; they were considered to be arterioles or venules (medium-sized vessels) when wider than 20 μm. The diameter of oval vessels was calculated as the half sum between the largest and the shortest dimension. To obtain the values of vascular density per area unit (0.33 mm^2^), both capillaries and medium-sized vessels were separately counted in six different fields of the sections that showed the highest vessel density. To quantify the inflammatory response per area unit (0.33 mm^2^), the intensity staining of CD3-positive cells was counted in ten different fields of the sections from the border zone. Image-Pro Plus software (Media Cybernetics, http://www.mediacy.com) was employed to perform counts and density calculations.

### Statistical analysis

Quantitative data are expressed as mean ± SEM. Except for the Peak/TTP data, which were analysed by the paired Student's *t* test, statistical analysis between two groups was performed by the unpaired Student's *t* test. Analysis between three or more groups was performed by one-way anova, followed by Tukey's test for comparisons between groups. A value of *P* ≤ 0.05 was considered statistically significant.

## Results

### Characterization, adhesion and viability of MSCs grown on HYAFF®11

**Fig. 1:**
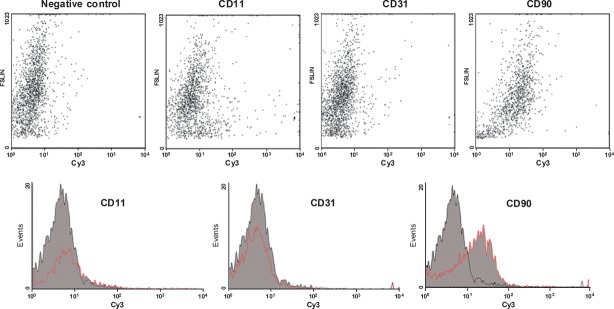
Flow cytometry analysis of pig MSC superficial antigens. Flow cytometry analysis shows that pig bone marrow mononuclear adherent cells stained negative for CD31 and CD11, which are markers of endothelial cells and monocytes/macrophages respectively. On the contrary, most cells stained positive for CD90, a superficial antigen that is detectable in MSCs. The negative control was obtained using the Cy3-conjugated antibody in the absence of the primary antibody.

Observation at the fluorescence microscopy (image not shown) revealed that MSCs stained negative for CD11, CD31 and MPF, while they expressed CD90, a superficial molecule present in MSCs that have been isolated from different animal species, including pigs 27, 28. These results were confirmed by flow cytometry analysis (Fig. 1).

**Fig. 2:**
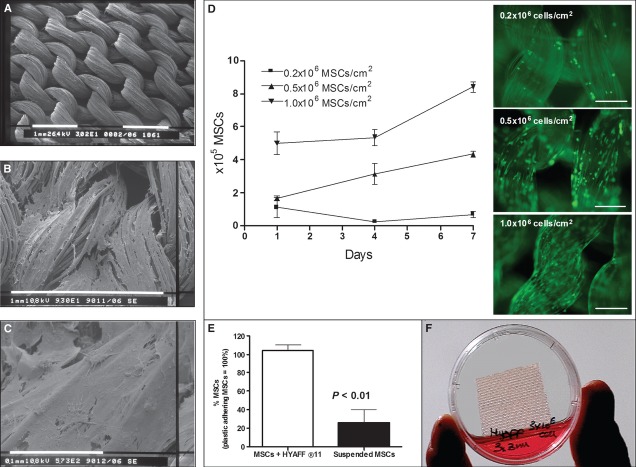
Morphological analysis and viability of MSCs seeded on HYAFF®11. The hyaluronan-based scaffold was analysed by SEM which revealed (**a**) the regular texture of HYAFF®11 without MSCs (white bar, 1 mm), (**b**) the adhesion of MSCs 1 week after seeding (white bar, 1 mm) and (**c**) their expansion on the fibres (white bar, 0.1 mm). Different MSC densities of seeding (0.2×, 0.5×, 1.0× 10^6^ cells/cm^2^) were initially tested to evaluate the best yield of cell adhesion and proliferation on HYAFF®11 (**d**). 1 × 10^6^ cells/cm^2^ gave the highest proliferation response evaluated by MTT analysis (each value represents the mean ± SEM of three separate experiments) and expressed as absolute number of viable MSCs. CFDA-loaded MSCs cultured on HYAFF®11 at the three different densities were also evaluated 1 week after seeding (right images; magnification = 100×; bar = 100 μm). Analysis also revealed (**e**) that MSCs cultured for 3 days on HYAFF®11 were about four times more viable than those grown in suspension (*P* < 0.01). These last values are expressed as mean percentage ± SEM (*n* = 3) of the viable MSCs with respect to those cultured on a plastic dish (100%). Finally, (**f**) the scaffold had a square-shaped appearance.

Scanning electron microscopy revealed the regular knitted texture of HYAFF®11 (Fig. 2a) and the strong adhesion of MSCs to the hyaluronan fibres embedded in a dense extracellular matrix (Fig. 2b and c). Three different densities (0.2×, 0.5×, 1.0× 10^6^ cells/cm^2^) were checked for MSC seeding on HYAFF®11. The highest yield (94.5%) was obtained with 1.0 × 10^6^ cells/cm^2^, whereas 0.2× and 0.5× gave a cell recovery of 82.0% and 84.5%, respectively. The corresponding cell viability and 1-week expansion on HYAFF®11 of CFDA-loaded MSCs at these three different densities are shown in Figure 2d. The best condition was obtained with the highest cell density which was chosen for transplantation to the infarct area. Using MTT analysis, the lowest cell density was used to compare the viability of the MSCs adhering to the fibres of HYAFF®11 with that of the MSCs in suspension. Figure 2e shows that MSCs cultured on HYAFF®11 for 3 days were about four times more viable than the same amount of cells grown in suspension (*P* < 0.01), suggesting that the process of anoikosis can be significantly blunted by growing MSCs onto this scaffold.

**Fig. 3:**
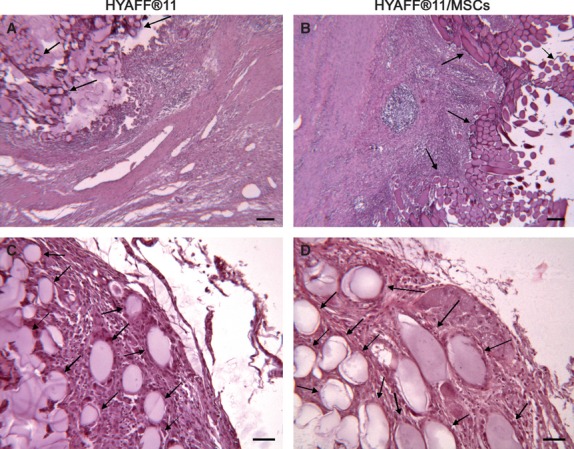
Morphology of HYAFF®11 fibres transplanted in the infarct area. The contour of the HYAFF®11 fibres from both HYAFF®11 and HYAFF®11/MSCs groups is oval or lengthened, according to the direction of the mesh. These samples were obtained 4 weeks after transplantation and were stained with haematoxylin and eosin. Scale bars: (**a** and **b**) 100 μm; (**c** and **d**) 50 μm.

The macroscopic square-shaped appearance of HYAFF®11 is shown in Figure 2f. In the HYAFF®11/MSCs group, the cells covered all the surface of the polymer (image not shown). Figure 3 shows the morphology of the HYAFF®11 fibres transplanted in the infarct area in both HYAFF®11 and HYAFF®11/MSCs groups.

The ability of MSCs to produce VEGF when supported by the hyaluronan-based fibres was also investigated. The amount of VEGF released under this condition was similar and not significantly different from that produced by the MSCs seeded on the plastic dish (*P* = 0.182) or suspended in their culture medium (*P* = 0.06). The concentrations of the released VEGF were as follows (pg/ml, *n* = 4): 690.6 ± 119.7, 655.7 ± 102.4 and 549.6 ± 72.1 respectively (data not shown).

### Blood perfusion of the infarcted heart

**Fig. 4:**
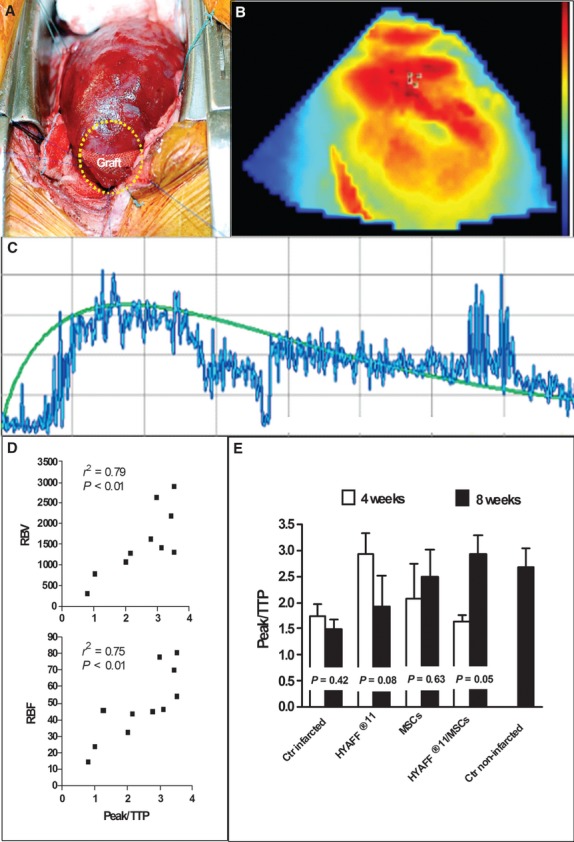
CEUS of the infarcted heart. (**a**) The figure shows the scaffold during its engraftment inside a pouch created by elevating a superficial flap of the epicardium of the infarcted area (yellow circle), 4 weeks after the coronary ligation. The scaffold was put on the centre of the infarct region, covering more than 50% of this area. (**b**) The blood flow was visualized by coloured tones fading from red to blue, corresponding to areas of high and low perfusion respectively. (**c**) The figure shows a representative slope of the 1-min. passage of the high-density particles through the elective area of investigation in a non-infarcted pig heart. (**d**) The ratio between Peak and TTP (Peak/TTP) positively correlated either with RBV or RBF (*P* < 0.01, *n* = 10). (**e**) The bar graph compares the Peak/TTP ratio measured 4 weeks after coronary ligation just before grafting with that obtained in the same pig 8 weeks after coronary ligation. Peak/TTP did not significantly change in the groups tested with the exception of the HYAFF®11/MSCs group whose value at week 8 was about two times that at week 4 (*P* = 0.05). The Peak/TTP ratio of the HYAFF®11/MSCs group was the same of that measured in a healthy pig of the same strain and weight 8 weeks after stabulation. Ctr infarcted, *n* = 5; HYAFF®11, *n* = 3; MSCs, *n* = 3; HYAFF®11/MSCs, *n* = 3; Ctr non-infarcted, *n* = 5.

The CEUS of the heart (Fig. 4 and Supporting Information) allowed the evaluation of the perfusion of the infarct region. The following parameters were determined by Qontrast software (Fig. 4c):

- the peak per cent (Peak), which represents the highest value of perfusion monitored for 1 min.;
- the time to peak (TTP), which is the time (sec.) lasting from the beginning of the CEUS to the achievement of the peak;
- the regional blood volume (RBV), which corresponds to the blood volume monitored for 1 min.;
- the regional blood flow (RBF), which corresponds to the blood flow monitored for 1 min.

Through a further elaboration of these parameters, one more index was considered the ratio between %Peak and TTP (Peak/TTP). This was very effective for evaluating the degree of perfusion of the investigated cardiac region because it strictly correlated (*P* < 0.01) with either RBV or RBF (Fig. 4d). Figure 2e shows the Peak/TTP ratio calculated under the different experimental conditions by comparing the values obtained 4 weeks after coronary ligation (just before graft transplantation) with those obtained in the same pig 8 weeks after coronary ligation. The Peak/TTP ratio did not significantly change in the untreated infarcted group or the HYAFF®11 and MSCs groups, but it increased about twofold in the HYAFF®11/MSCs group (*P* = 0.05). Moreover, the Peak/TTP ratio of the HYAFF®11/MSCs group was comparable with that of non-infarcted, healthy swine measured in the same region of the heart.

### *In vivo* tracking of BrdU-labelled MSCs

**Fig. 5:**
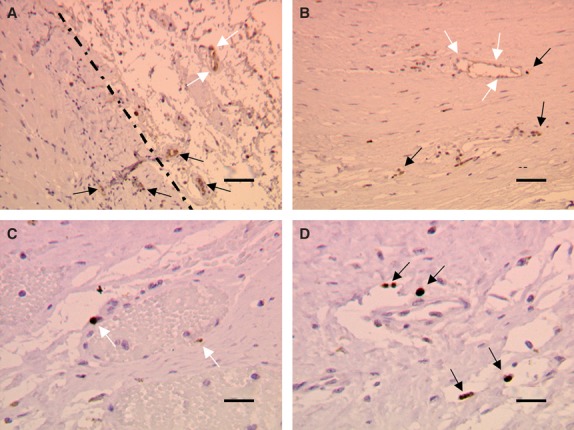
Localization of BrdU-labelled MSCs. In the myocardial preparations, the dark brown elements identified the nuclei of MSCs pre-loaded with BrdU before implantation. (**a**) BrdU-labelled MSCs were mainly present in the infarct region and in the border zone (dotted line), whereas few of them moved to the healthy remote regions of the heart (data not shown). (**b**–**d**) Many BrdU^+^MSCs were localized at the level of vessels structures, just near them (black arrows), or inside the vessel wall (white arrows). Scale bars: (a and b) 25 μm, (c and d) 10 μm.

Four weeks after grafting, BrdU^+^ MSCs were mainly identified in the infarct area and in the border zone of both MSCs and HYAFF®11/MSCs groups (Fig. 5), whereas very few BrdU^+^ MSCs moved to the healthy remote regions of the heart (image not shown). Many labelled MSCs were localized near the vessels structures (Fig. 5, black arrows) and inside the vessel wall (Fig. 5, white arrows).

### Vessel density

**Fig. 6:**
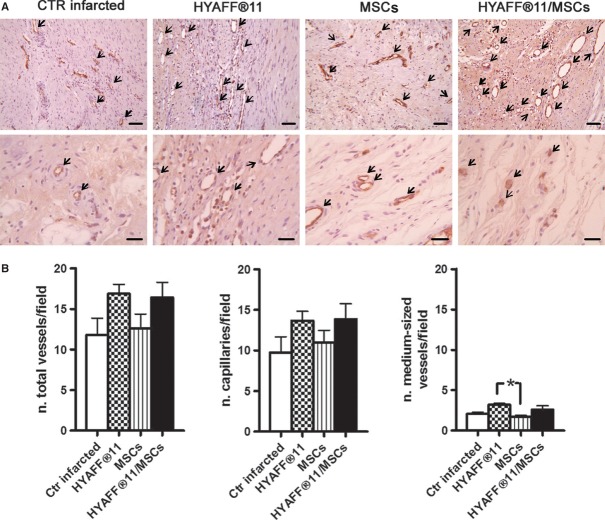
Vascularization of the infarct area. (**a**) The blood vessels were identified in the infarct region as circular/oval structures stained with vWF as indicated by the arrows (upper bars, 50 μm; lower bars, 25 μm). (**b**) The quantitative analysis was performed with all the representative regions of the infarct area (both centre region and border zone). In the bar graph, the number of total vessels corresponds to the sum of capillaries and medium-sized vessels. The number of medium-sized vessels significantly increased in the HYAFF®11 group with respect to the MSCs group (*P* < 0.05). Ctr infarcted, *n* = 5; HYAFF®11, *n* = 3; MSCs, *n* = 3; HYAFF®11/MSCs, *n* = 4.

Blood vessels were identified in the infarct region as circular/oval structures lined by one or more vWF^+^ cells (Fig. 6a). The number of medium-sized vessels was significantly increased in the HYAFF®11 group relative to the MSCs group (*P* < 0.05; Fig. 6b). The number of capillaries and total vessels (capillaries + medium-sized vessels) was apparently increased in both groups treated with HYAFF-11, but this number did not reach statistical significance with respect to the untreated infarct group.

### Inflammatory reaction

**Fig. 7:**
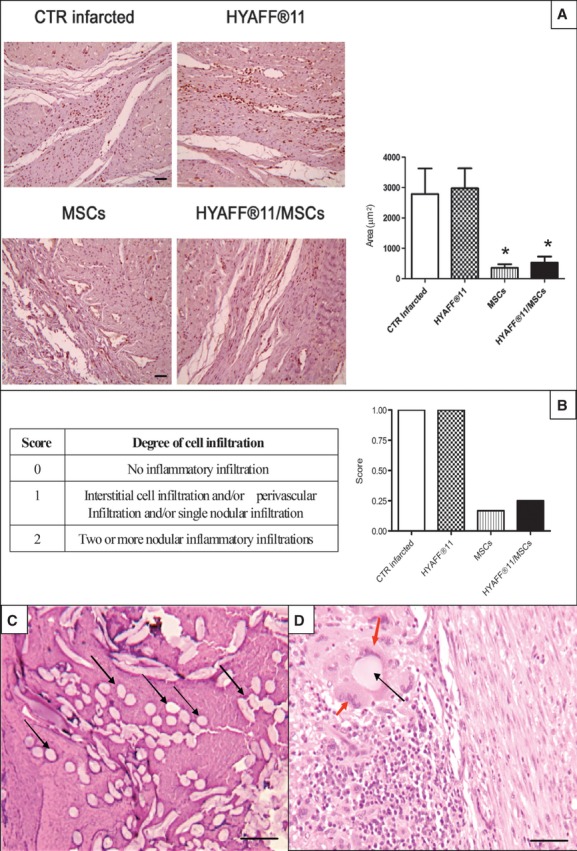
Inflammatory reaction and related score of the infarct area. (**a**) The micrographs show the inflammation process expressed as area stained with CD3-positive inflammatory cells (lymphocytes), whereas the bar graph shows the statistical difference among groups. The transplantation of MSCs, with or without HYAFF®11, almost completely reduced the density of cellular infiltrate. Scale bars: 100 μm. *P < 0.05 *versus* both Ctr infarcted and HYAFF®11 groups. (**b**) The quantification of the inflammatory reaction was confirmed by attributing a score from 0 to 2, according to the degree of cell infiltration (table inside). (**c** and **d**) A foreign body reaction was clearly activated close to the fibres of HYAFF®11 (black arrows) 4 weeks after transplantation. Scale bars: (c) 100 μm; (d) 50 μm. Macrophages and giant cells were found only around the fibres of the scaffold (red arrows). Ctr infarcted, *n* = 5; HYAFF®11, *n* = 3; MSCs, *n* = 3; HYAFF®11/MSCs, *n* = 4.

The degree of the inflammation process was expressed either as number of infiltrated cells (Fig. 7b) or as the area stained with CD3-positive inflammatory cells (Fig. 7a). Using both criteria, the hearts of the HYAFF®11 group showed an overall degree of inflammation similar to that of the untreated infarct group. The score of both the HYAFF®11 and HYAFF®11/MSCs groups was calculated taking the foreign body reaction that was bordered only on the fibres of the scaffold (Fig. 7c and d) into account. The transplantation of MSCs, with or without HYAFF®11, almost completely reduced the infarction-dependent cellular infiltrate (Fig. 7a and b), confirming the notion that MSCs can exert an important immunosuppressive action.

### Extracellular matrix and cardiomyocyte morphology

**Fig. 8:**
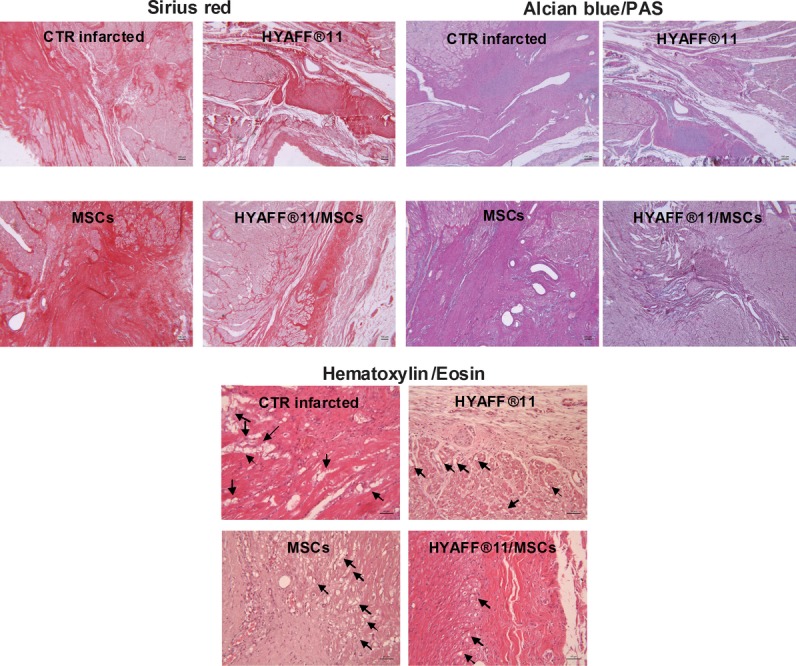
Extracellular matrix and cardiomyocyte morphology. Micrographs are representative of the border zone of the infarct area. The HYAFF®11 and HYAFF®11/MSCs groups showed the lesser amount of collagen types I and III (Sirius Red), corresponding to a reduced fibrosis, when compared with the MSCs and untreated infarct groups (scale bar, 100 μm). The HYAFF®11/MSCs group showed also the highest presence of proteoglycans in the extracellular matrix (Alcian Blue) with respect to the other groups (scale bar, 100 μm). Although nearby the fibrotic area vacuolization and degeneration of cardiac cells (arrows) were present in all the groups (Haematoxylin and Eosin), this finding was attenuated in the HYAFF®11/MSCs group (scale bar, 50 μm).

At the border zone, fibrosis was characterized using the highly hydrophylic Sirius Red dye that stains collagen I and III fibres; as seen in Figure 8, the HYAFF®11 and HYAFF®11/MSCs groups showed the lesser amount of red stained collagen when compared with the MSCs and untreated infarct groups. After Alcian Pas staining on sections from the same areas the HYAFF®11/MSCs group showed an intensely alcianophilic extracellular matrix. In the corresponding haematoxylin and eosin stained sections, morphological changes were seen in cardiomyocyte facing the fibrotic scar. Although cardiomyocyte hypertrophy was not observed, vacuolization and degeneration of cardiac cells was a finding in all the investigated groups even though the HYAFF®11/MSCs group was less affected.

## Discussion

This study illustrates the effectiveness of restoring blood perfusion in the fibrous region of the infarcted myocardium after transplantation of autologous MSCs with a hyaluronan-based scaffold. The myocardial perfusion in pigs was analysed using CEUS. The most sensitive parameter used was the Peak/TTP ratio because it correlated with both RBV and RBF. This index was restored only in the infarct region of the hearts treated with the complete construct HYAFF®11/MSCs, doubling the value detected in the same pig before transplantation. The Peak/TTP ratio of the infarct area of the HYAFF®11/MSCs group was even comparable with that of the non-infarcted healthy animals. To assess the degree of vascularization induced by the different treatments scheduled in this study, both capillary and medium-sized vessel densities were measured in the infarct area of the explanted hearts. In the HYAFF®11 group the number of medium-sized vessels was increased compared with the MSCs group, whereas no statistical difference was observed between the other groups. To explain why blood perfusion increased in the HYAFF®11/MSCs group with respect to the other groups in the presence of similar amount of vessels, we suggest that in this scaffold-cell construct MSCs have synergically contributed to improving blood flow by blunting inflammation thereby decreasing the oedema-dependent compression exerted on the small/medium vessels. Moreover, it should be taken into account that the *in vitro* viability of the MSCs adhering to the scaffold was higher than that of MSCs in suspension, which supports the hypothesis that a higher number of MSCs can survive ischaemic stress when attached to HYAFF®11 rather than injected alone. In addition, the MSCs ability to produce VEGF, one of the most powerful angiogenic factors, was not precluded by their adhesion to HYAFF®11.

On the other hand, the mechanism by which HYAFF®11 increased the vessel density could be related to the gradual fragmentation of its fibres into small-sized hyaluronate oligosaccharides 15. Indeed, hyaluronan with 6–10 residues has been described as: (i) promoting the proliferation of HUVEC, (ii) inducing angiogenesis in the chicken chorioallantoic membrane assay and (iii) increasing VEGF mRNA levels in HUVEC 29. In our condition, the localized foreign inflammatory reaction around the fibres of HYAFF®11 could have contributed to the acceleration of the breakdown of hyaluronan into smaller fragments. It has also been documented that HYAFF®11, when soaked in a physiological solution and in the absence of hydrolytic enzymes, releases about 90% of its benzyl alcohol within 20 days 16. This data support the hypothesis that a number of fibres could be already fragmented 4 weeks after scaffold transplantation in *in vivo* conditions, despite the initial resistance of HYAFF®11 to hyaluronidase degradation 16. These small hyaluronan oligomers are able to activate vascular endothelial cells through the CD44 and RHAMM receptors which, in turn, stimulate the ERK-dependent angiogenic process 30.

Although we did not observe any consistent increase in the Peak/TTP ratio in the MSCs group, positive effects on blood perfusion after the injection of bone marrow MSCs in the infarcted area of the pig heart, without any polymeric support, have been reported by other investigators 31–33. In our study, the lack of improvement in blood flow after the transplantation of MSCs only could result from differences in experimental protocols. For instance, rather than grafting MSCs soon after the induction, we waited 4 weeks to avoid the acute phase of the inflammatory response, to decrease the risk of arrhythmias and to await the formation of the scar. Autologous MSCs were also injected by Liu *et al*. in a swine model of cardiac infarction, leading to an increase in vessel density 27. Again, we suppose that this finding seems to contrast the absence of neovascularization observed in our MSCs group because the induction of MI was substantially different. In the infarction model of Liu *et al*., the coronary ligation was followed by reperfusion and MSCs were injected just after reperfusion. Moreover, vessel density was assessed a short period of time after grafting and no inherent information was given 4 weeks after cell injection. Moreover, in that study, the data concerning myocardial perfusion were not provided; therefore, we cannot conclude that the increased vascularization was really effective in enhancing regional blood flow.

In our study, the biocompatibility of HYAFF®11 in the infarct region of the heart was also satisfactory, as already demonstrated in other tissues 16. Indeed, despite a typical foreign body reaction that was confined to the hyaluronan fibres, an amount of infiltrated cells, including CD3-positive lymphocytes, comparable with that observed in the untreated infarcted group were found in the remaining myocardial region. That the presence of MSCs in the construct attenuated this inflammatory response is probably owing to their innate immunosuppressive properties 34.

Apart from the inflammatory response, the native tissue positively interacted with the HYAFF®11/MSCs construct modifying the extracellular matrix with a reduced presence of collagene and increased amount of proteoglycans. The border-zone cardiomyocytes also reacted favourably to the graft as a lower degree of cellular damage was found.

In conclusion, these findings demonstrate that in the scar following a myocardial infarction the transplantation of autologous bone marrow MSCs with a hyaluronan-based knitted scaffold completely restores blood perfusion and strongly attenuates the inflammation process. These positive effects are superior to those obtained by grafting MSCs alone or the scaffold itself.

## Supporting information

**Video S1** CEUS of a pig heart.
